# Oestrogen receptors regulate vascular β₁- and β₃-adrenoceptor expression and shape sex-specific adrenergic control of vascular tone

**DOI:** 10.1007/s00424-026-03182-z

**Published:** 2026-05-22

**Authors:** Kristin Riedel, Andreas Deussen, Birgit Zatschler, Carmen Hentsche, Bianca Müller, Stephan Speier, Christian Reeps, Sems M. Tugtekin, Klaus Matschke, Irakli Kopaliani

**Affiliations:** 1 Institute of Physiology, Faculty of Medicine Carl Gustav Carus, TUD Dresden University of Technology, Fetscherstraße 74, 01307 Dresden, Germany; 2 Paul-Langerhans-Institute Dresden of the Helmholtz Zentrum München, Faculty of Medicine and University Hospital Carl Gustav Carus of TUD Dresden University of Technology, Helmolz Zentrum München, 85764 Neuherberg, Germany; 3 Division of Vascular and Endovascular Surgery, Department of Visceral, Thoracic and Vascular Surgery, Faculty of Medicine and University Hospital Carl Gustav Carus, TUD Dresden University of Technology, Fetscherstraße 74, 01307 Dresden, Germany; 4 Department of Cardiac Surgery, Herzzentrum Dresden, TUD Dresden University of Technology, Fetscherstraße 76, 01307 Dresden, Germany

**Keywords:** β-adrenoceptors, Vascular tone, Oestrogen receptor, Endothelial function, Sex differences

## Abstract

Sex differences in vascular adrenergic responsiveness contribute to differential regulation of vascular tone. Female vessels exhibit enhanced β-adrenergic relaxation compared with males, but the role of oestrogen receptors in maintaining this phenotype and the capacity of sex hormone exposure to reprogram vascular β-adrenoceptor expression remain incompletely understood. Thoracic aorta and mesenteric arteries from Wistar Kyoto rats were examined following pharmacological oestrogen receptor inhibition or cross-sex hormone treatment. Vascular reactivity was assessed using wire myography, and β₁-, β₂-, and β₃-adrenoceptor mRNA expression was quantified by qPCR. Oestrogen receptor blockade with fulvestrant enhanced norepinephrine-induced vasoconstriction and reduced β-adrenergic relaxation in both conduit and resistance arteries. These functional alterations were accompanied by an approximately 50% reduction in β₁- and β₃-adrenoceptor mRNA expression, whereas β₂ expression remained unchanged. Conversely, oestrogen treatment in males attenuated vasoconstriction, enhanced β-adrenergic relaxation, and increased β₁- and β₃-adrenoceptor expression, while testosterone treatment in females had no effects. Despite pronounced vascular changes, arterial blood pressure remained unaltered. Analysis of human aorta, carotid, iliac, and mammary arteries revealed consistently higher β₁- and β₃-adrenoceptor mRNA expression in women compared with men. Oestrogen receptor inhibition reduces vascular β₁- and β₃-adrenoceptor expression and impairs β-adrenergic vasodilation, thereby shifting adrenergic vascular responses towards enhanced constriction. Conversely, oestrogen exposure reprogrammes the male vascular phenotype towards a female-like β-adrenergic profile. These findings identify an oestrogen receptor–dependent β₁/β₃-adrenoceptor axis as a conserved mechanism underlying sex-specific regulation of vascular tone.

## Introduction

Neuronal regulation of vascular tone is a fundamental physiological mechanism by which the sympathetic nervous system regulates arterial pressure and adequate tissue perfusion. Adrenergic stimulation induces vasoconstriction primarily through activation of α-adrenoceptors in vascular smooth muscle cells, while concomitant activation of β-adrenoceptors can evoke vasorelaxation [19, 33, 34]. The net vascular response to sympathetic activation therefore reflects the balance between constrictor α-adrenergic and dilator β-adrenergic signalling, which differs between vascular beds and may be altered under pathological conditions [8, 18, 19, 27]. Because vascular tone is a major determinant of total peripheral resistance, alterations in adrenergic signalling have direct consequences for arterial blood pressure regulation [16, 28, 29].

Accumulating evidence indicates that adrenergic control of vascular tone differs between sexes. In humans, norepinephrine-induced vasoconstriction is generally more pronounced in men, whereas women exhibit attenuated constrictor responses and enhanced vasorelaxation to β-adrenergic stimulation [20, 21]. Studies using forearm blood flow measurements have demonstrated stronger β-adrenergic vasodilator responses in women compared with men, both under resting conditions and during sympathetic activation [17, 21]. These sex-specific differences in adrenergic vascular reactivity are conserved at least across humans and rats and suggest intrinsic differences in vascular adrenergic signalling pathways that may contribute to sex-dependent patterns of blood pressure regulation and cardiovascular risk [6, 9, 13, 23, 24, 29].

The vascular endothelium plays a central role in the modulation of adrenergic vessel tone through the release of vasoactive mediators, most prominently nitric oxide (NO) [12]. Activation of endothelial β-adrenoceptors has been shown to stimulate NO production, thereby counteracting α-adrenoceptor-mediated smooth muscle contraction during adrenergic stimulation [4, 11, 26]. Among β-adrenoceptor subtypes, β₁- and β₃-adrenoceptors have been implicated in endothelial NO-dependent vasorelaxation in rat and human vessels, whereas β₂-adrenoceptors are thought to act predominantly at the level of vascular smooth muscle cells [3, 27, 31, 35].

In previous studies, we demonstrated that sex-specific differences in adrenergic vascular reactivity are closely linked to differential expression and function of endothelial β-adrenoceptors. Female vessels exhibit higher expression of β₁- and β₃-adrenoceptors, resulting in enhanced NO-dependent vasorelaxation to β-adrenergic stimulation and a blunted vasoconstrictor response to norepinephrine [1]. These differences are observed in rat experimental models and human mammary arteries and are abolished by endothelial removal or inhibition of NO, underscoring the functional relevance of endothelial β-adrenergic signalling [1]. Subsequently, we proposed that the oestrogen is a key upstream regulator of endothelial β₁- and β₃-adrenoceptor expression and function, whereas progesterone and testosterone do not exert similar effects [30]. Together, these findings established an oestrogen–endothelial β₁/β₃–NO signalling axis as a major determinant of sex-specific adrenergic control of vascular tone.

Despite these advances, critical questions remain unresolved. In particular, the contribution of oestrogen receptors to the maintenance of endothelial β-adrenoceptor expression and function in vivo has not been fully elucidated. Furthermore, it is unclear to what extent endothelial β-adrenergic regulation is determined by biological sex itself or by the prevailing sex hormone environment. Specifically, it remains unknown whether changes in sex hormone exposure are sufficient to reprogram adrenergic vascular responsiveness, vascular tone regulation, and blood pressure control, or whether these mechanisms are irreversibly fixed by sex-specific vascular organization. To address these gaps in knowledge, we examined the impact of pharmacological inhibition of oestrogen receptors and altered sex hormone exposure on β-adrenergic vessel tone regulation and arterial pressure.

## Methods

### Animal experiments

All animal experiments were approved by the responsible local authorities (approval number: DD24.1-5131/449/11) and conducted in accordance with the ARRIVE guidelines and with the European Directive 2010/63/EU for animal experiments.

Wistar Kyoto rats were obtained from Charles River Laboratories at three weeks of age and housed under standard laboratory conditions with free access to food and water. After three weeks of adaptation, experimental treatments were initiated at six weeks of age.

For pharmacological inhibition of oestrogen receptors, female rats received fulvestrant (ICI 182,780; Sigma-Aldrich) administered at a dose of 5 mg/kg body weight via subcutaneous route once a week for 6 weeks [5, 7, 15]. Vehicle-treated animals received the corresponding vehicle solution.

For cross-hormone substitution male rats received oestrogen (2 mg/kg body weight), and female rats received testosterone (2 mg/kg body weight). Hormones were administered orally by mixing the respective compound into hazelnut cream (Nutella^®^, Ferrero). Vehicle-treated control groups received hazelnut cream without hormone supplementation. Hormonal substitution was performed twice weekly for a total duration of six weeks. The selection of the vehicle and the dose of the hormones was based on our previous work, as well as other reported studies [30, 32].

In all animal experiments, six rats per experimental group were used. At twelve weeks of age, animals were euthanized under deep anaesthesia induced by intraperitoneal injection of Ketamine/Xylazine in a dose of 100/10 mg/kg and vessels were collected for subsequent functional and molecular analyses.

### Measurement of arterial blood pressure

Systolic and diastolic blood pressure was measured in conscious animals using a non-invasive tail-cuff system. Rats were placed in restraining tubes and acclimatised to the procedure prior to measurements. Blood pressure recordings were obtained using a tail-cuff plethysmography device as previously described [2]. Multiple consecutive measurements were recorded for each animal, and the mean value was used for subsequent analysis. All measurements were performed under standardized conditions at the end of the treatment period.

### RNA isolation and quantitative real-time PCR

For analysis of β-adrenoceptor expression, total RNA was isolated from thoracic aortic tissue and mesenteric arteries [1, 10, 30]. Vessel segments were snap-frozen, homogenized, and processed using TRIzol reagent according to the manufacturer’s instructions. RNA concentration and purity were assessed spectrophotometrically.

Complementary DNA was synthesized from equal amounts of total RNA using a reverse transcription kit following standard protocols. Quantitative real-time PCR was performed using gene-specific primers for β₁-, β₂-, and β₃-adrenoceptors and a fluorescent detection system. Amplification was carried out under optimized cycling conditions, and melting curve analysis was used to confirm specificity of the PCR products. Gene expression levels were normalized to housekeeping genes and expressed relative to control groups using the comparative Ct method. Primer sequences and validation procedures were identical to those established previously [1, 10, 30].

### Assessment of vascular reactivity using wire myography

Vascular function was assessed ex vivo using a Mulvany wire myograph system [1, 14, 25, 30]. Thoracic aorta and mesenteric arteries were carefully dissected, freed from surrounding adipose tissue, and cut into ring segments of standardized length.

Part of the experiments on the vessel rings was performed after removal of endothelium. For this, a cotton wire was introduced into the lumen of the vessel segment and gently moved back and forth several times [22, 30].

Vessel rings were mounted on stainless steel wires in organ chambers containing physiological solution, continuously aerated and maintained at 37 °C. After an equilibration period, vessels were normalized to an internal circumference corresponding to a defined transmural pressure, as described previously [1, 14, 25, 30].

Contractile capacity was verified using a potassium-enriched solution. Adrenergic vasoconstriction was assessed by dose-dependent application of norepinephrine. For vasorelaxation studies, vessels were preconstricted with norepinephrine or potassium-enriched solution, followed by a dose-dependent addition of β-adrenergic agonist (Isoprenaline, BRL 37344). Part of the experiments were performed in the presence of combined β_1_ (CGP 20712, 1 µmol/l) and β_2_ (ICI 118.551, 100 nmol/l) inhibitors, or β_3_ adrenoceptor blockade (SR 59230 A, 1 µmol/l). Responses were recorded continuously and expressed as changes in wall tension or as percentage relaxation relative to preconstriction.

### Human vessel preparation

Human vascular tissue was obtained from patients undergoing surgery after written informed consent and approval by the local ethics committee (EK 307-12-2015). Segments of aorta, common carotid artery, iliac artery, and mammary artery of women and men aged 48–56 years were collected intraoperatively and immediately placed in a protective preservation solution to maintain tissue integrity [1, 14, 30].

After removal of surrounding adipose tissue, vessel segments were snap-frozen in liquid nitrogen and stored at − 80 °C until further processing. Total RNA was subsequently isolated from these vascular samples, and β₁-, β₂-, and β₃-adrenoceptor mRNA expression levels were quantified as previously performed [1, 2, 30].

### Materials

17β-Oestradiol, testosterone propionate, norepinephrine, and fulvestrant (ICI 182,780) were purchased from Sigma-Aldrich (St. Louis, MO, USA). Isoprenaline (cat. no. 1747), the selective β₃-adrenoceptor agonist BRL 37,344, and the selective β-adrenoceptor antagonists CGP 20,712 (β₁), ICI 118,551 (β₂), and SR 59,230 A (β₃) were obtained from Tocris Bioscience (Bristol, UK). Acetylcholine (ACh) and sodium nitroprusside (SNP) were purchased from Sigma-Aldrich. All other chemicals were of analytical grade and obtained from standard commercial suppliers.

### Statistical analysis

Statistical analyses were performed using GraphPad Prism version 10 (GraphPad Software, San Diego, CA, USA). Concentration–response curves were analysed by two-way analysis of variance (ANOVA). When appropriate, multiple comparisons were adjusted using Bonferroni post hoc correction.

Comparisons of maximal vasoconstriction and vasorelaxation between experimental groups were analyzed using two-way ANOVA followed by Bonferroni post hoc correction. Data distribution was assessed using the Shapiro–Wilk test prior to application of parametric statistical analyses. In all experiments, ‘n’ refers to the number of animals analysed and therefore represents biological replicates.

For human vascular mRNA expression data, comparisons between women and men were performed using an unpaired two-tailed Student’s t-test. Data are presented as mean ± SEM. A P value < 0.05 was considered statistically significant.

## Results

### Oestrogen receptor inhibition enhances adrenergic vasoconstriction and blunts β-adrenergic relaxation in aorta

**Fig. 1:**
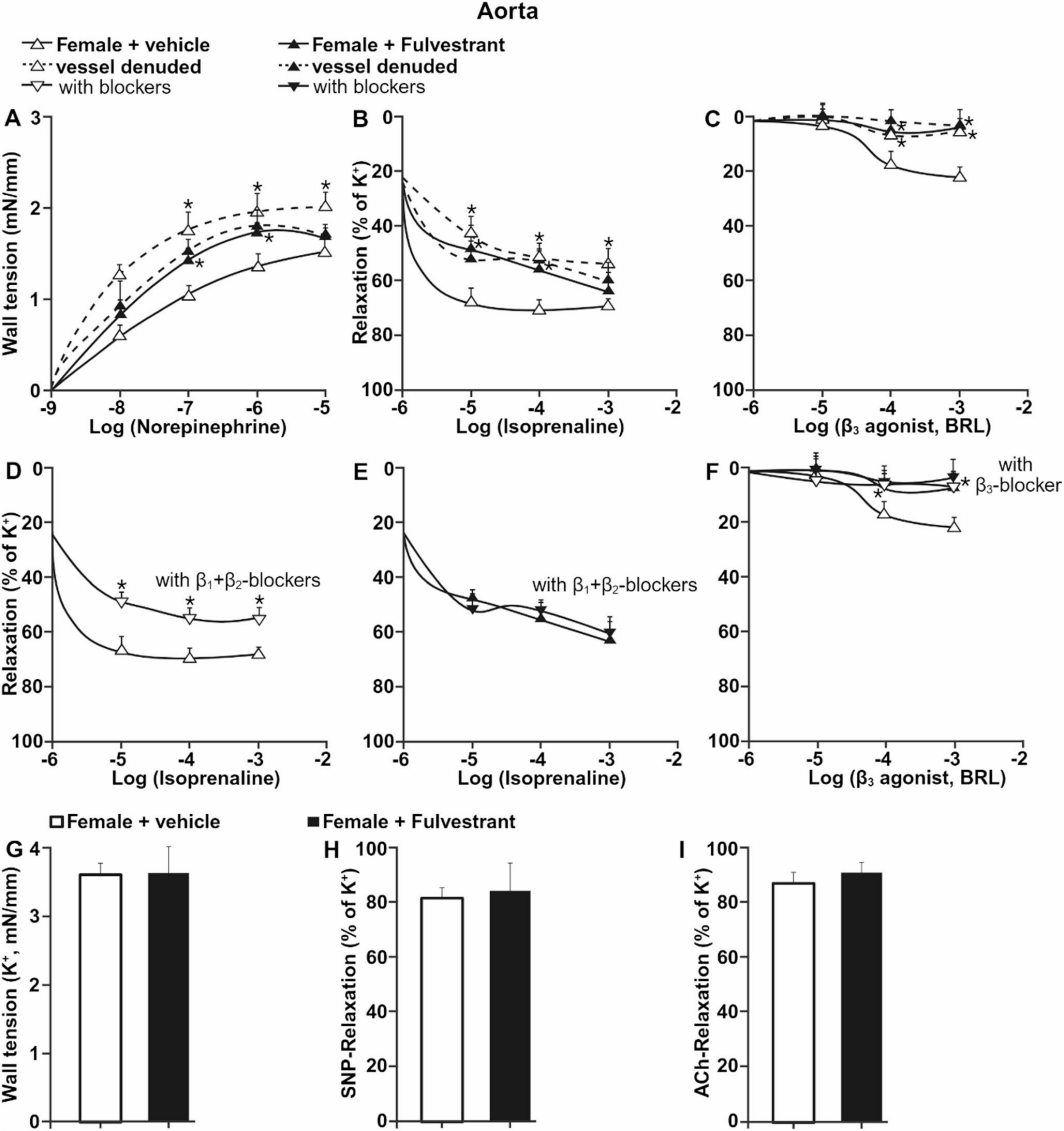
Oestrogen receptor blockade enhances adrenergic vasoconstriction and impairs β-adrenergic relaxation in aorta. (**A**) Norepinephrine-induced increase in wall tension in aortic rings from vehicle- and fulvestrant-treated female rats under endothelium-intact and endothelium-denuded conditions. (**B**) Isoprenaline-induced relaxation in preconstricted aortic rings. (**C**) Relaxation in response to selective β₃-adrenoceptor stimulation. (**D–E**) Isoprenaline-induced relaxation in the presence of combined β₁- and β₂-adrenoceptor blockade in vehicle-treated (**D**) and fulvestrant-treated (**E**) females. (**F**) β₃-adrenoceptor-mediated relaxation including in the presence of β₃ blockade. (**G**) Maximal contraction induced by potassium-enriched solution (KCl). (**H**) Endothelium-independent relaxation to sodium nitroprusside (SNP). (I) Endothelium-dependent relaxation to ACh. Data are presented as mean ± SEM (*n* = 6). *P < 0.05 vs. vehicle

To determine the impact of oestrogen receptor inhibition on adrenergic vascular reactivity, aortic rings from vehicle- and fulvestrant-treated female rats were examined ex vivo (Fig. 1). In response to cumulative norepinephrine stimulation, aortas from fulvestrant-treated females developed greater wall tension compared with vehicle-treated controls (Fig. 1A). Removal of the endothelium markedly increased norepinephrine-induced wall tension in aortas from vehicle-treated females. In contrast, endothelial removal did not further augment vasoconstriction in aortas from fulvestrant-treated females (Fig. 1A). As a result, endothelium-denuded vessels from both groups exhibited similar levels of vasoconstriction.

Isoprenaline-induced vasorelaxation was reduced in aortas from fulvestrant-treated females compared with vehicle-treated controls (Fig. 1B). Endothelial removal attenuated isoprenaline-mediated relaxation in vehicle-treated aortas but had no further effect in fulvestrant-treated vessels (Fig. 1B). Stimulation with the selective β₃-adrenoceptor agonist induced approximately 20% relaxation in aortas from vehicle-treated females, whereas relaxation was nearly abolished in fulvestrant-treated vessels (Fig. 1C). Removal of the endothelium eliminated β₃-mediated relaxation in vehicle-treated aortas but did not alter responses in fulvestrant-treated vessels (Fig. 1C).

In vehicle-treated aortas, combined β₁- and β₂-adrenoceptor blockade reduced isoprenaline-induced relaxation (Fig. 1D). In contrast, β₁/β₂ blockade did not significantly modify isoprenaline responses in aortas from fulvestrant-treated females (Fig. 1E). Similarly, β₃-adrenoceptor-mediated relaxation was diminished by β₃ blockade in vehicle-treated vessels. In fulvestrant-treated aortas, β₃-adrenoceptor stimulation failed to induce measurable relaxation, and therefore β₃ blockade did not further modify vascular responses (Fig. 1F).

Maximal contractions induced by potassium-enriched solution were similar between vehicle- and fulvestrant-treated groups (Fig. 1G). For this reason, β-adrenergic relaxations were normalized to potassium-induced preconstriction, as norepinephrine elicited different contractile amplitudes between groups.

Endothelium-independent relaxation induced by sodium nitroprusside did not differ between groups (Fig. 1H). Likewise, acetylcholine-induced relaxation was similar in vehicle- and fulvestrant-treated aortas in presence of endothelium (Fig. 1I).

### Oestrogen receptor inhibition alters β-adrenergic reactivity in mesenteric arteries

**Fig. 2:**
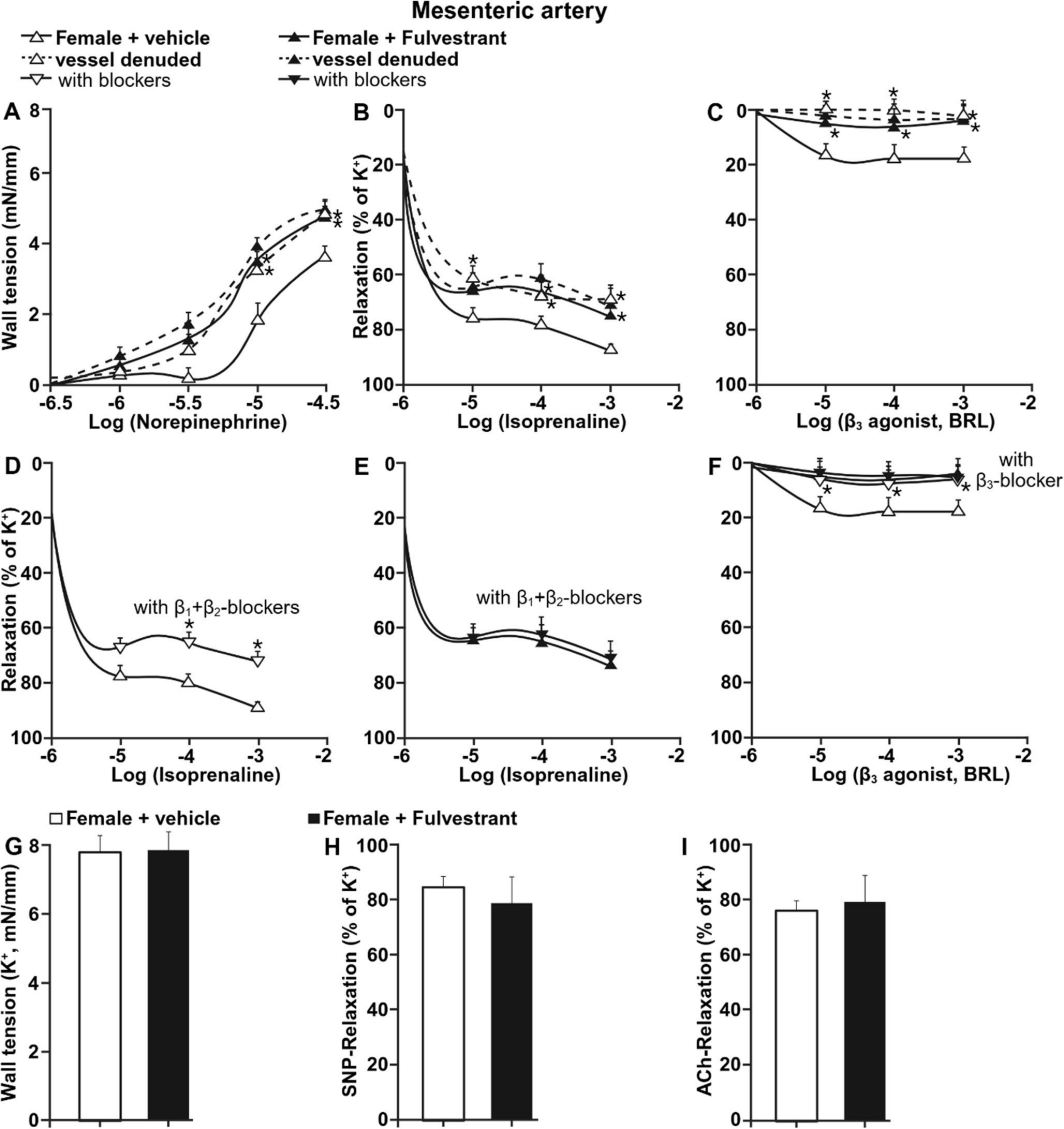
Oestrogen receptor inhibition alters β-adrenergic reactivity in mesenteric arteries. (**A**) Norepinephrine-induced contraction in mesenteric arteries from vehicle- and fulvestrant-treated female rats. (**B**) Isoprenaline-induced relaxation. (**C**) Relaxation in response to selective β₃-adrenoceptor stimulation. (**D–E**) Isoprenaline-induced relaxation in the presence of combined β₁- and β₂-adrenoceptor blockade. (**F**) β₃-adrenoceptor-mediated relaxation including in the presence of β₃ blockade. (**G**) Maximal KCl-induced contraction. (**H**) Endothelium-independent relaxation to SNP. (**I**) Endothelium-dependent relaxation to ACh. Data are presented as mean ± SEM (*n* = 6). *P < 0.05 vs. vehicle

To determine whether the effects observed in aorta extend to resistance vessels, mesenteric arteries from vehicle- and fulvestrant-treated female rats were examined under similar experimental conditions (Fig. 2). Norepinephrine-induced vasoconstriction was greater in mesenteric arteries from fulvestrant-treated females compared with vehicle-treated controls (Fig. 2A). Removal of the endothelium increased wall tension in vehicle-treated arteries but did not further enhance vasoconstriction in fulvestrant-treated vessels.

Isoprenaline induced marked relaxation in mesenteric arteries from vehicle-treated females, reaching maximal relaxation of approximately 90%. In contrast, maximal relaxation was reduced to approximately 70% in arteries from fulvestrant-treated females (Fig. 2B). Endothelial removal attenuated isoprenaline-induced relaxation in vehicle-treated arteries, whereas it did not further reduce relaxation in fulvestrant-treated vessels.

Stimulation with the selective β₃-adrenoceptor agonist resulted in ~ 20% relaxation in mesenteric arteries from vehicle-treated females. This response was absent in arteries from fulvestrant-treated animals (Fig. 2C). Removal of the endothelium abolished β₃-mediated relaxation in vehicle-treated arteries but did not alter responses in fulvestrant-treated vessels.

Combined β₁- and β₂-adrenoceptor blockade reduced isoprenaline-induced relaxation in mesenteric arteries from vehicle-treated females, whereas in fulvestrant-treated vessels β₁/β₂ blockade did not significantly modify isoprenaline responses (Fig. 2D–E). β₃-adrenoceptor-mediated relaxation was nearly abolished by β₃ blockade in vehicle-treated arteries. In contrast, mesenteric arteries from fulvestrant-treated females did not exhibit measurable relaxation in response to β₃-adrenoceptor stimulation, and therefore β₃ blockade did not further modify vascular responses (Fig. 2F).

Maximal contraction induced by potassium-enriched solution did not differ between groups. Endothelium-independent relaxation to sodium nitroprusside and endothelium-dependent relaxation to acetylcholine were similar between groups in presence of endothelium (Fig. 2G-I).

### Oestrogen receptor inhibition reduces β₁- and β₃-adrenoceptor mRNA expression in aorta and mesenteric arteries

**Fig. 3:**
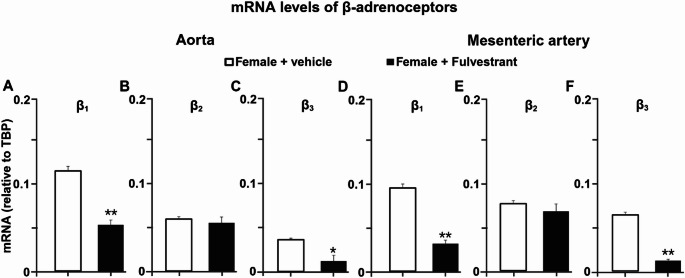
Fulvestrant reduces β₁- and β₃-adrenoceptor mRNA expression in aorta and mesenteric arteries. (**A–B**) Relative mRNA expression of β₁-, β₂-, and β₃-adrenoceptors in thoracic aorta (**C–D**) Relative mRNA expression in mesenteric arteries from vehicle- and fulvestrant-treated female rats. Data are presented as mean ± SEM (*n* = 6). *P < 0.05, **P < 0.01 vs. vehicle

To determine whether the functional alterations induced by fulvestrant were associated with changes in β-adrenoceptor expression, mRNA levels of β₁-, β₂-, and β₃-adrenoceptors were quantified in thoracic aorta and mesenteric arteries (Fig. 3). In both vascular beds, fulvestrant treatment reduced β₁-adrenoceptor mRNA expression compared with vehicle-treated females (approximately 50% in both aorta and mesenteric arteries). Similarly, β₃-adrenoceptor mRNA expression was reduced in fulvestrant-treated animals, reaching roughly half of control levels in both vessel types. In contrast, β₂-adrenoceptor mRNA expression did not differ between vehicle- and fulvestrant-treated females in either aorta or mesenteric arteries.

### Cross-sex hormone exposure selectively modifies adrenergic vascular reactivity in aorta

**Fig. 4:**
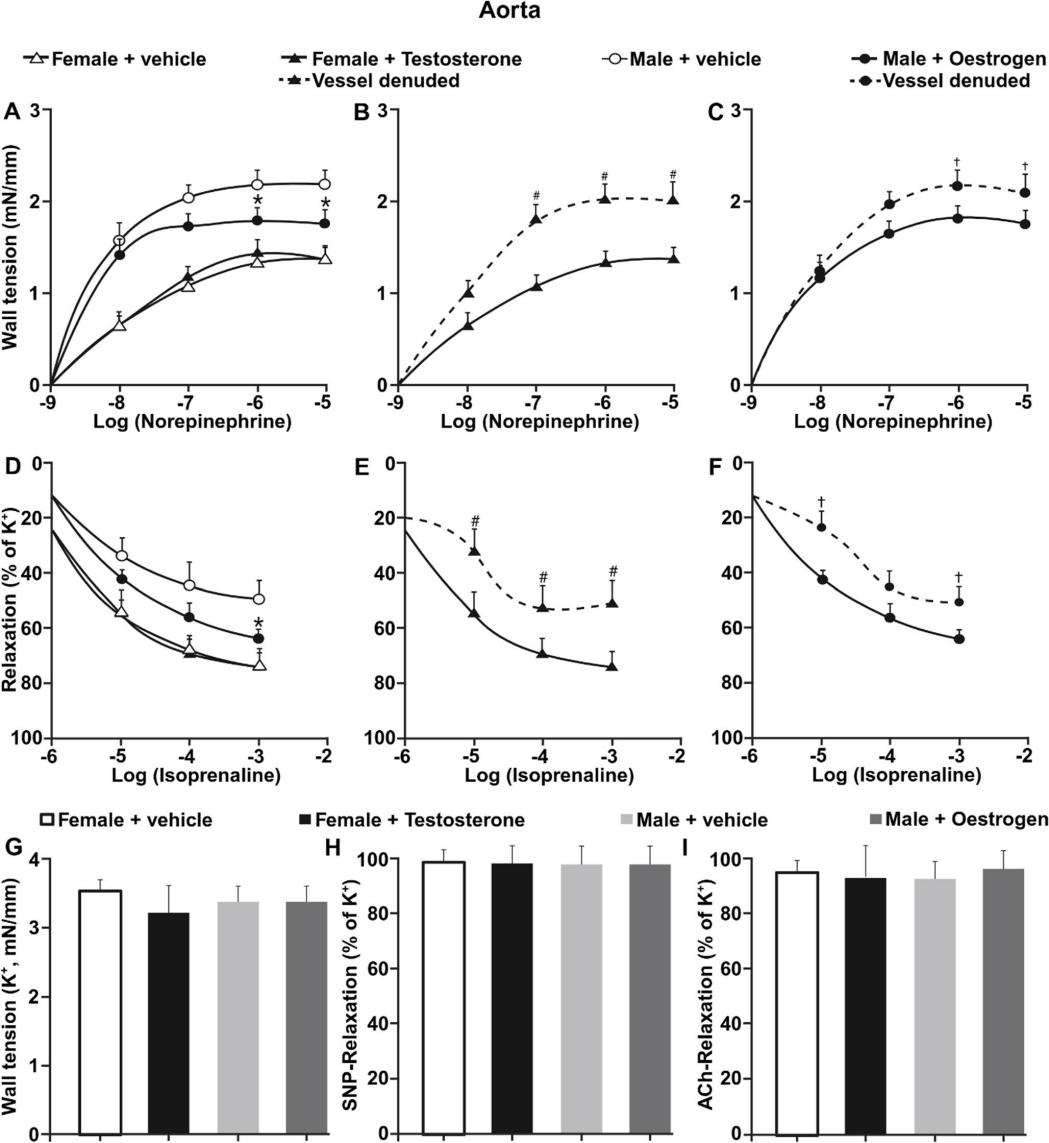
Cross-sex hormone exposure modulates adrenergic reactivity in aorta. (**A**) Norepinephrine-induced wall tension in aortic rings from vehicle-treated females, testosterone-treated females, vehicle-treated males, and oestrogen-treated males (**B–C**) Norepinephrine responses under endothelium-intact and endothelium-denuded conditions in cross-treated groups. (**D**) Isoprenaline-induced relaxation in all experimental groups. (**E–F**) Isoprenaline-induced relaxation in endothelium-intact and endothelium-denuded vessels. (**G**) Maximal KCl-induced contraction. (**H**) Endothelium-independent relaxation to SNP. (**I**) Endothelium-dependent relaxation to ACh. Data are presented as mean ± SEM (*n* = 6). *P < 0.05 vs. male + vehicle group; #*P* < 0.05 vs. female + testosterone; †*P* < 0.05 vs. male + oestrogen

To determine whether alterations in systemic sex hormone milieu modify adrenergic vascular responsiveness, aortic rings from four groups were examined: vehicle-treated females, testosterone-treated females, vehicle-treated males, and oestrogen-treated males (Fig. 4).

Consistent with previous observations [30], norepinephrine-induced wall tension was greater in aortas from vehicle-treated males compared with vehicle-treated females (Fig. 4A). Testosterone treatment in females did not alter norepinephrine-induced vasoconstriction compared with vehicle-treated females. In contrast, oestrogen treatment in males reduced wall tension development compared with vehicle-treated males.

To assess endothelial contribution, norepinephrine responses were examined in endothelium-intact and endothelium-denuded vessels. In testosterone-treated females, removal of the endothelium enhanced norepinephrine-induced wall tension (Fig. 4B). Similarly, in oestrogen-treated males, endothelial removal enhanced norepinephrine-induced vasoconstriction (Fig. 4C).

Isoprenaline-induced relaxation differed between sexes under vehicle conditions, with aortas from vehicle-treated females exhibiting stronger maximal relaxation than those from vehicle-treated males (Fig. 4D). Testosterone treatment in females did not modify isoprenaline-induced relaxation, whereas oestrogen treatment in males increased maximal relaxation compared with vehicle-treated males. In both cross-treated groups, endothelial removal attenuated β-adrenergic relaxation compared with endothelium-intact vessels (Fig. 4E, F).

Maximal potassium-induced contraction, sodium nitroprusside-induced relaxation, and acetylcholine-mediated relaxation were similar across experimental groups in presence of endothelium (Fig. 4G-I).

### Cross-sex hormone exposure reprograms β-adrenergic vascular function and receptor expression

**Fig. 5:**
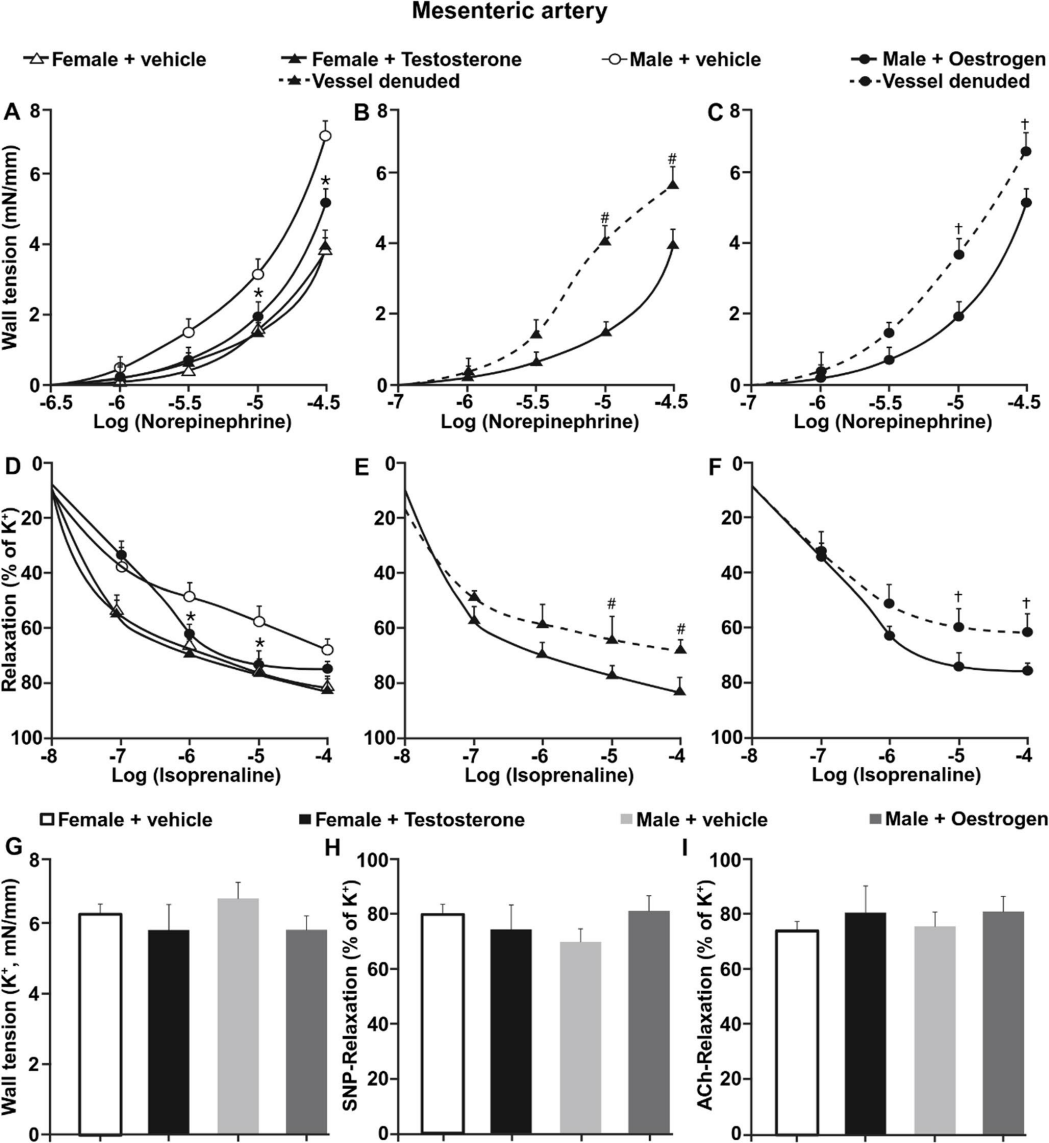
Cross-sex hormone exposure alters adrenergic responses in mesenteric arteries. (**A**) Norepinephrine-induced concentration–response curves in endothelium-intact mesenteric arteries. Norepinephrine-induced contraction following endothelial removal in female (**B**) and male vessels (**C**). (**D**) Isoprenaline-induced relaxation under endothelium-intact conditions. Isoprenaline-induced relaxation following endothelial removal in female (**E**) and male (**F**) rats. (**G**) Maximal contraction induced by potassium-enriched solution (KCl). (**H**) Endothelium-independent relaxation to sodium nitroprusside (SNP). (**I**) Endothelium-dependent relaxation to acetylcholine (ACh). Data are presented as mean ± SEM (*n* = 6). *P < 0.05 vs. male + vehicle group; #*P* < 0.05 vs. female + testosterone; †*P* < 0.05 vs. male + oestrogen

Functional responses in mesenteric resistance arteries mirrored those observed in aorta (Fig. 5). Under vehicle conditions, norepinephrine-induced contraction was greater in males than in females. Testosterone treatment did not alter contractile responses in females, whereas oestrogen treatment attenuated vasoconstriction in males (Fig. 5A). β-adrenergic relaxation was enhanced in females compared with males and was increased in oestrogen-treated males compared to respective vehicle group, but remained unchanged in testosterone-treated females (Fig. 5D). Endothelial removal increased norepinephrine-induced constriction and reduced isoprenaline-induced relaxation in both cross-treated groups (Fig. 5B, C, E, F). Control responses to potassium, sodium nitroprusside, and acetylcholine were not different between groups (Fig. 5G-I).

**Fig. 6:**
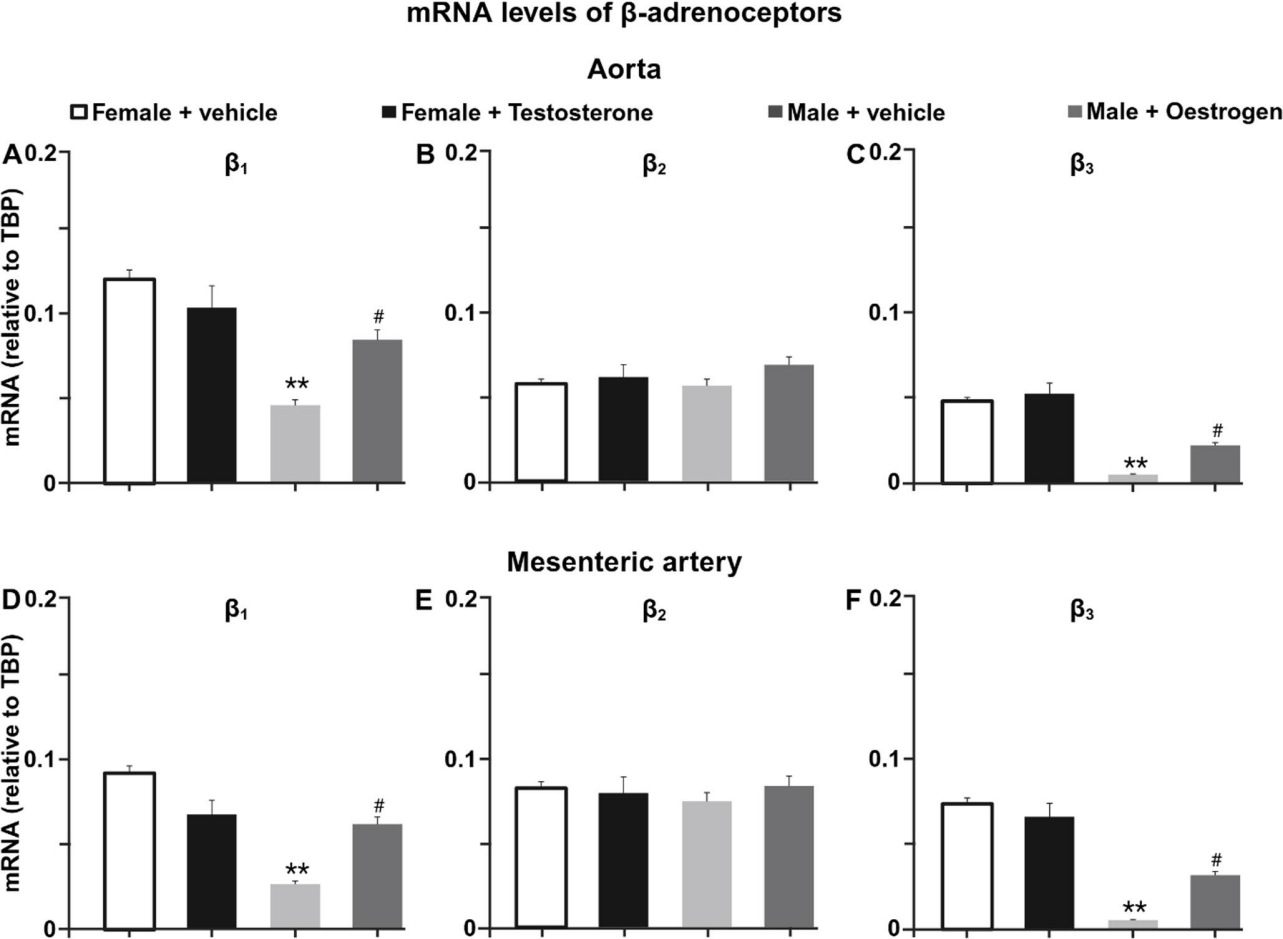
Cross-sex hormone exposure alters β₁- and β₃-adrenoceptor expression in rat vessels. (**A–B**) Relative β₁-, β₂-, and β₃-adrenoceptor mRNA expression in thoracic aorta (**C–D**) Corresponding expressions in mesenteric arteries. Data are presented as mean ± SEM (*n* = 6). **P < 0.01 vs. females + vehicle; ^#^*P* < 0.05 vs. male + vehicle

Molecular analyses revealed a pattern consistent with the functional findings (Fig. 6). In both aorta and mesenteric arteries, oestrogen treatment in males increased β₁- and β₃-adrenoceptor mRNA expression compared with vehicle-treated males, whereas testosterone treatment in females did not alter β₁- or β₃-adrenoceptor mRNA levels compared with vehicle-treated females. β₂-adrenoceptor mRNA expression remained unchanged across all experimental groups and vascular beds.

### Arterial blood pressure remains unchanged following oestrogen receptor inhibition and cross-sex hormone exposure

Vehicle- and fulvestrant-treated female rats exhibited similar arterial blood pressure values (115/75 ± 5 mmHg vs. 118/74 ± 6 mmHg, *n* = 6), corresponding to mean arterial pressures of approximately 88 and 89 mmHg, respectively. Likewise, cross-sex hormone exposure did not significantly alter arterial blood pressure in either sex. Testosterone treatment in females (114/78 ± 6 mmHg; MAP ≈ 90 mmHg) and oestrogen treatment in males (125/80 ± 4 mmHg; MAP ≈ 95 mmHg) resulted in blood pressure values comparable to their respective vehicle-treated controls (116/76 mmHg ± 5; MAP ≈ 89 mmHg in females and 124/80 ± 7 mmHg; MAP ≈ 95 mmHg in males). However, under control conditions, male rats exhibited higher arterial blood pressure compared with females. Thus, despite marked alterations in vascular adrenergic responsiveness and β-adrenoceptor expression, systemic arterial pressure remained largely unchanged across experimental interventions.

### Sex differences in β-adrenergic receptor expression in human vessels

**Fig. 7:**
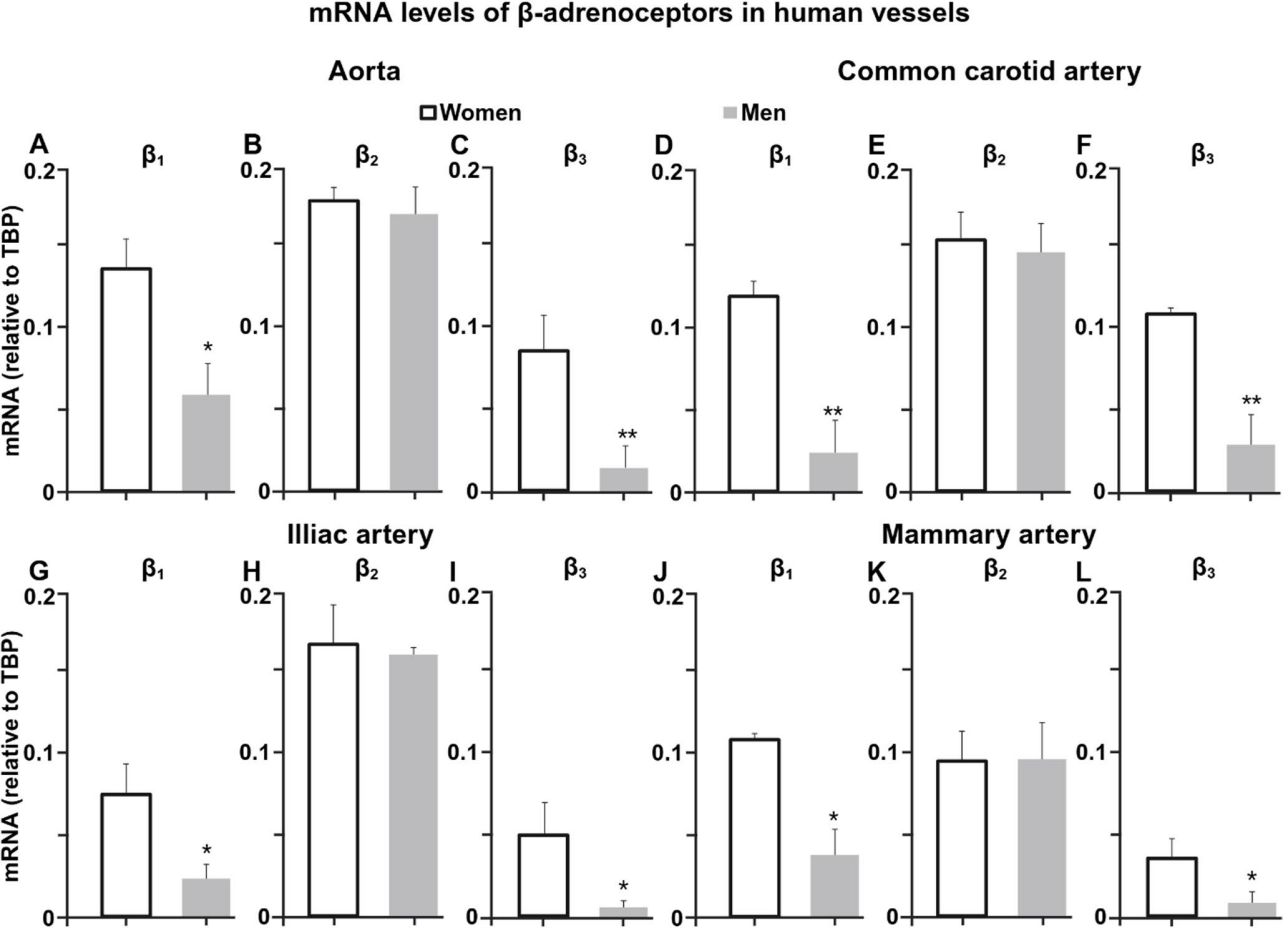
Sex differences in β-adrenoceptor mRNA expression in human vascular tissue. Relative mRNA expression of β₁-, β₂-, and β₃-adrenoceptors in human (**A**) aorta, (**B**) common carotid artery, (**C**) iliac artery, and (**D**) mammary artery obtained from women and men. Data are presented as mean ± SEM (*n* = 7–21). *P < 0.05, **P < 0.01 vs. women

The levels of β-adrenoceptor mRNA were quantified in human aorta, common carotid artery, iliac artery and mammary artery (Fig. 7). Across all vessel types examined, β₁-adrenoceptor mRNA expression was higher in women compared with men, with male vessels showing approximately 50% lower levels. A similar pattern was observed for β₃-adrenoceptor expression, which was reduced in men compared with women across all vascular beds. In contrast, β₂-adrenoceptor mRNA expression did not differ between women and men in any vessel analysed.

## Discussion

The present study identifies oestrogen receptor as a central determinant of vascular β-adrenergic responsiveness and provides evidence that the systemic sex hormone milieu selectively reprograms vascular tone regulation. Pharmacological inhibition of oestrogen receptors with fulvestrant, a compound known to preferentially target oestrogen receptor α (ERα), enhanced norepinephrine-induced vasoconstriction and attenuated β-adrenergic relaxation in both conduit and resistance arteries. These functional alterations were paralleled by a selective reduction in β₁- and β₃-adrenoceptor mRNA expression, whereas β₂-adrenoceptor expression remained unchanged. Conversely, oestrogen exposure in male rats attenuated vasoconstriction, enhanced β-adrenergic relaxation, and increased β₁- and β₃-adrenoceptor expression, while testosterone exposure in females had minimal effects. Importantly, similar sex differences in β₁- and β₃-adrenoceptor expression were observed in multiple human vascular beds. Together, these findings extend our previous work and support the concept that oestrogen receptor–dependent regulation maintains a β₁/β₃-mediated vasodilatory axis that contributes to sex-specific control of vascular tone during adrenergic stimulation [1, 30].

In our earlier work, we demonstrated that female vessels exhibit enhanced β-adrenergic relaxation and higher endothelial β₁- and β₃-adrenoceptor expression compared with males, and that these differences are abolished by endothelial removal or nitric oxide inhibition. We further identified oestrogen as a key upstream regulator of endothelial β₁- and β₃-adrenoceptor expression [1, 30]. The present findings directly build upon those observations by showing that pharmacological blockade of oestrogen receptors in intact female animals reproduces a male-like vascular phenotype, characterised by enhanced vasoconstriction and impaired β-adrenergic relaxation.

Importantly, endothelial removal markedly reduced β-adrenergic relaxation in vehicle-treated females but did not further attenuate relaxation in fulvestrant-treated vessels. This indicates that fulvestrant largely abolishes the endothelial component of β-adrenergic vasodilation. Residual vasorelaxation observed in denuded vessels is consistent with preserved smooth muscle β-adrenoceptor-mediated relaxation, likely involving β₂-adrenoceptors. Consistent with this interpretation, fulvestrant treatment did not alter acetylcholine-mediated relaxation or sodium nitroprusside responses, demonstrating preserved global endothelial integrity and smooth muscle responsiveness. The selective downregulation of β₁- and β₃-adrenoceptor mRNA, without changes in β₂ expression, further supports the notion that oestrogen receptor activity preferentially maintains an endothelial β₁/β₃-mediated vasodilatory pathway rather than exerting a generalised effect on endothelial or vascular smooth muscle β₂-adrenoceptor and its function. Together, these findings support the concept of an oestrogen receptor–dependent endothelial β₁/β₃-adrenoceptor axis governing sex-specific β-adrenergic responsiveness.

Across experimental conditions, functional alterations in vascular tone closely paralleled changes in β₁- and β₃-adrenoceptor expression. Under control conditions, female vessels displayed enhanced β-adrenergic relaxation and reduced vasoconstriction compared with males, corresponding to higher β₁- and β₃-adrenoceptor mRNA levels. These findings are consistent with our previous experimental data [2, 30] and with human forearm and conduit artery studies demonstrating attenuated sympathetic vasoconstriction and enhanced β-adrenergic vasodilation in women [17, 20, 21].

The stability of β₂-adrenoceptor expression across all interventions further highlights the specific contribution of β₁- and β₃-adrenoceptors to the female vascular phenotype [1, 30]. While β₂-adrenoceptors have classically been considered central mediators of β-adrenergic vasodilation, the present results support a more prominent role for β₁- and β₃-adrenoceptors in sex-dependent vascular modulation [1, 30].

Modification of the systemic sex hormone milieu revealed a marked alteration in sex-dependent vascular function. Oestrogen treatment in males attenuated vasoconstriction, enhanced β-adrenergic relaxation, and increased β₁- and β₃-adrenoceptor expression, thereby shifting the male vascular phenotype toward a female-like pattern. In contrast, testosterone treatment in females did not significantly alter vascular reactivity or receptor expression. These findings extend our previous demonstration that oestrogen is the decisive upstream regulator of endothelial β₁/β₃-adrenoceptor expression and function [30]. They suggest that the vascular β-adrenergic phenotype is primarily oestrogen-dependent and that androgen exposure alone is insufficient to induce reciprocal reprogramming. Importantly, the responsiveness of male vasculature to oestrogen indicates that the adult vascular system retains the capacity for hormonal modulation of adrenergic receptor expression.

From a translational perspective, these findings may have implications for individuals undergoing gender-affirming hormone therapy. In biological males receiving oestrogen treatment, vascular β-adrenergic responsiveness may shift toward a female-like phenotype, potentially altering sympathetic vascular control. Conversely, testosterone administration in biological females may not equivalently reverse oestrogen-dependent vascular signalling pathways. While the present data do not address clinical outcomes, they suggest that oestrogen-driven regulation of β₁- and β₃-adrenoceptors may represent a mechanistic component of potential vascular adaptation during hormonal transition [30].

A decline in circulating oestrogen levels is a defining feature of menopause and is associated with increased cardiovascular risk and altered vascular function [28, 29]. Our previous work mimicking menopause in ovariectomized animals demonstrated reduced β₁- and β₃-adrenoceptor expression and impaired β-adrenergic relaxation, which were restored by oestrogen supplementation [30]. The present findings extend this concept by showing that pharmacological oestrogen receptor blockade in intact females similarly reduces β₁- and β₃-adrenoceptor expression and enhances vasoconstriction.

These results support the notion that loss of oestrogen signalling may shift the vascular adrenergic balance toward increased constrictor tone [1, 20, 21, 30]. However, while oestrogen replacement restores β-adrenergic receptor expression in experimental models, clinical hormone replacement therapy remains complex and cannot be inferred as a direct therapeutic strategy based solely on β-adrenoceptor expression patterns. Vascular β-adrenoceptor regulation may represent only one component of cardiovascular risk, while systemic hormone therapy entails broader physiological effects.

Despite substantial modulation of vascular adrenergic responsiveness and receptor expression, arterial blood pressure remained unchanged across experimental interventions. This dissociation underscores the complexity of blood pressure regulation, which integrates neural, renal, and vascular mechanisms across multiple organ systems and is tightly controlled by systemic feedback mechanisms [16]. In contrast, isolated vessel preparations lack these compensatory regulatory loops. The present data suggest that oestrogen-dependent modulation of β-adrenergic receptor expression influences vascular reactivity without necessarily altering physiological arterial pressure under basal conditions. Such changes may become more relevant under conditions of heightened sympathetic activation, ageing or cardiovascular stress [29].

Analysis of human aorta, carotid, iliac, and mammary arteries revealed consistently higher β₁- and β₃-adrenoceptor mRNA expression in women compared with men, while β₂ expression remained unchanged. The preservation of this pattern across rat and human vessels and across multiple vascular beds supports the translational relevance of the experimental findings and suggests that sex-dependent β₁/β₃-adrenoceptor regulation represents a conserved feature of vascular physiology relevant for humans [1, 20, 21, 30]. These differences may contribute to sex-specific responses to sympathetic stimulation and potentially influence the vascular effects of β-adrenergic pharmacotherapy [17, 20, 21, 35]. The concordance between experimental and human data strengthens the physiological significance of the oestrogen-dependent β₁/β₃-adrenoceptor axis.

Collectively, the present study demonstrates that oestrogen receptor activity is a key determinant of vascular β₁- and β₃-adrenoceptor expression and β-adrenergic relaxation. Pharmacological inhibition of oestrogen receptors enhanced vasoconstriction and diminished β-adrenergic responsiveness, whereas oestrogen exposure in males shifted vascular function towards a female-like phenotype.

While the present experiments do not delineate the precise intracellular mechanisms or receptor subtype-specific pathways involved, the consistent functional and transcriptional changes observed across vascular beds strongly support a regulatory role of oestrogen receptor activity in maintaining endothelial β₁/β₃-mediated vasodilation.

The conservation of β₁- and β₃-adrenoceptor sex differences in human vessels further underscores the physiological relevance of this mechanism. Together, these findings identify an oestrogen receptor–dependent β₁/β₃-adrenoceptor axis as an important contributor to sex-specific regulation of vascular tone.

### Limitations

The present study has several limitations that should be acknowledged. First, the regulation of vascular β₁- and β₃-adrenoceptors was assessed at the mRNA level and was not complemented by direct protein-level quantification. Although the observed transcriptional changes closely paralleled the functional vascular responses, additional protein-based validation would further strengthen the mechanistic interpretation of the findings. However, reliable detection of vascular β-adrenoceptor proteins, particularly β₃-adrenoceptors, remains technically challenging due to limited antibody specificity and relatively low receptor abundance in vascular tissue.

Second, detailed information regarding menopausal status was not consistently available for all female human tissue donors. Given the age range of the cohort, peri- or postmenopausal hormonal status may have influenced vascular β-adrenoceptor expression. Nevertheless, higher β₁- and β₃-adrenoceptor expression was consistently observed in vessels obtained from women across multiple vascular beds, supporting the presence of preserved sex-dependent differences in human vascular tissue.

## Data Availability

All data supporting the findings of this study are available within the paper.
